# *Vibrio parahaemolyticus* strengthens their virulence through modulation of cellular reactive oxygen species *in vitro*

**DOI:** 10.3389/fcimb.2014.00168

**Published:** 2014-12-17

**Authors:** Shimaa S. El-Malah, Zhenquan Yang, Maozhi Hu, Qiuchun Li, Zhiming Pan, Xinan Jiao

**Affiliations:** ^1^Jiangsu Key Laboratory of Zoonosis/Jiangsu Co-Innovation Center for the Prevention and Control of Important Animal Infectious Diseases and Zoonoses, Yangzhou UniversityYangzhou, China; ^2^College of Food Science and Engineering, Yangzhou UniversityYangzhou, China; ^3^Testing Center, Yangzhou UniversityYangzhou, China

**Keywords:** *vibrio parahaemolyticus*, caco-2, ros, *in vitro*, infection, modulation

## Abstract

*Vibrio parahaemolyticus (Vp)* is one of the emergent food-borne pathogens that are commensally associated with various shellfish species throughout the world. It is strictly environmental and many strains are pathogenic to humans. The virulent strains cause distinct diseases, including wound infections, septicemia, and most commonly, acute gastroenteritis, which is acquired through the consumption of raw or undercooked seafood, especially shellfish. *Vp* has two type three secretion systems (T3SSs), which triggering its cytotoxicity and enterotoxicity via their effectors. To better understand the pathogenesis of *Vp*, we established a cell infection model *in vitro* using a non-phagocytic cell line. Caco-2 cells were infected with different strains of *Vp* (pandemic and non-pandemic strains) and several parameters of cytotoxicity were measured together with adhesion and invasion indices, which reflect the pathogen's virulence. Our results show that *Vp* adheres to cell monolayers and can invade non-phagocytic cells. It also survives and persists in non-phagocytic cells by modulating reactive oxygen species (ROS), allowing its replication, and resulting in complete cellular destruction. We conclude that the pathogenicity of *Vp* is based on its capacities for adhesion and invasion. Surprisingly's; enhanced of ROS resistance period could promote the survival of *Vp* inside the intestinal tract, facilitating tissue infection by repressing the host's oxidative stress response.

## Background

*Vibrio parahaemolyticus* (*Vp*) is a good example of emergent food pathogens. It is a Gram-negative halophilic bacterium found in estuarine, marine, and coastal environments. It occupies a variety of niches, and can exist in a free-swimming state using a single polar flagellum or with sessile attachment to inert or animate surfaces, such as suspended particulate matter, zooplankton, fish, and shellfish (McCarter, 1999). Since its discovery in the middle of the last century (Fujino et al., 1953), it has become renowned as a leading cause of seafood-derived food poisoning throughout the world. Where the virulent strains are transmitted by the consumption of raw or undercooked seafood, it is a common cause of acute gastroenteritis (Newton et al., 2012). Although, the diarrhea it causes is self-limiting, it can also cause septicemia, which can be life threatening to patients with pre existing medical conditions (Su and Liu, 2007). In Japan, *Vp* is responsible for 20–30% of all cases of food poisoning (Alam et al., 2002) and is considered a common cause of seafood-borne illnesses in many Asian countries (Yu et al., 2013). In the United States, it has also become the leading agent of human gastroenteritis associated with seafood consumption (Newton et al., 2012).

*Vp* is a natural resident of estuarine and marine environments, and only some strains have proven to be pathogenic (Oliver and Kaper, 2007). Even when they lack the thermostable hemolysins of *Vp*, some strains remain pathogenic, indicating the existence of other virulence factors (Xu et al., 1994). In this context, Kanagawa phenomenon (KP)-negative clinical strains of *Vp* have shown the ability to produce a second hemolysin, TDH-related hemolysin (TRH) (Honda et al., 1988). Differences observed among the O3:K6 strains led to the definition of non-pandemic O3:K6 as those strains isolated in 1980–1990 in several Asian countries, including India, China, Japan, Thailand, and Bangladesh (Okuda et al., 1997; Matsumoto et al., 2000; Osawa et al., 2002; Wong et al., 2005).

The contribution of T3SSs to these activities is still unclear under conditions in which Thermostable direct hemolysin (TDH) is produced together with adherence factors, including the outer membrane, pili, lateral flagella, and cell-associated hemagglutinin (HA) (Nagayama et al., 1995). Since the discovery of *Vp* in Japan in 1950 and its global occurrence as a leading cause of seafood-derived food poisoning, there has been extensive research to understand the virulence of this pathogen and its effects on human as accidental host.

The lack of clarity about the pathogenic mechanism of *Vp* is one of the main problems facing researchers. The links between strains isolated from the environment, from seafood, and from human clinical isolates are poorly understood, in contrast to the other food-borne infections. *Vp* infections are not related to socioeconomic status, meteorological changes, quality of the water supply, or general sanitation conditions. Thus, the overall mechanism of the enteropathogenesis of *Vp* has not been fully clarified.

The Caco-2 cell line, derived from a moderately differentiated human colonic adenocarcinoma (Pinto et al., 1983), has been useful in evaluating the adherence and invasion of bacterial pathogens (Knutton et al., 1989).

Reactive oxygen species (ROS) is one of the early responses of host innate immunity which produced in reaction to microbial invaders. Free oxygen radicals (ROS) are highly toxic to pathogens and so utilized as a tool to prevent microorganisms' colonization in tissues (Circu and Aw, 2010), the importance of ROS for immune function could be exploited by “potential pathogens” which lead to host responses reduction and enhance survival and colonization of pathogens in their target host cells.

In this study, we undertook to establish a cell infection model to determine the cytotoxic effects of pandemic and non-pandemic clinical isolates of *Vp* to clarify its pathogenetic mechanisms *in vitro*.

## Materials and methods

### Bacterial strains and cell line

The *Vibrio parahaemolyticus* strains investigated were RIMD2210633 (KP positive, serotype O3:K6) (Nasu et al., 2000), Vp024 and Vp038 (pandemic isolates), and Vp005 and Vp029 (non-pandemic isolates). All strains kept in the Laboratory of Zoonoses and Immunology, Yangzhou University, China, were recovered from −70°C on TCBS medium, then maintained in MLB medium, and genotyped with specific primers. Caco-2 cells were used as the mammalian cell model for the experiment and were cultured in Dulbecco's Modified Eagle medium (DMEM; Gibco®, Grand Island, NY, USA) supplemented with 10% fetal bovine serum (FBS; Gibco®, Grand Island, NY, USA) plus 1% antibiotic Pen strep (Gibco®, Grand Island, NY, USA) at 37°C under 5% CO_2_ until fully confluent. All bacterial strains and the cell line used in this study are listed in Table 1. And the primers used in all PCRs are listed in Table 2.

**Table 1:** **Bacterial strains and cell line used in this study**.

**Strain (*Vp*)**	**Description**
RIMD2210633	Clinical isolate; KP positive; serotype O3:K6
Vp005	Non-pandemic; Clinical isolate; KP positive
Vp024	Pandemic; Clinical isolate; KP positive
Vp029	Non-pandemic; Clinical isolate; KP negative
Vp038	Pandemic; Clinical isolate; KP positive
Caco-2	Human colorectal adenocarcinoma cell line isolated in late 1970s from colon carcinoma tissue

**Table 2:** **Primers used for genotyping the strains**.

**Gene target**	**Primer sequence**	**Band size (bp)**	**References**
*tlh*	tlh-F:5′AAAGCGGATTATGCAGAAGCACTG 3′	450	Bej et al., 1999
	tlh-R:5′ GCTACTTTCTAGCATTTTCTCTGC 3′		
*tdh*	tdh-F:5′ ATATCCATGTTGGCTGCATTC 3′	513	Chao et al., 2009
	tdh-R:5′ TTATTGTTGATGTTTACATTCAAA A 3′		
*trh*	trh-F:5′ ATGAAACTAAAACTCTACTTTGC 3′	545	
	trh-R:5′ TTAATTTTGTGACATACATTCAT 3′		
*orf8*	orf8-F:5′GTTCGCATACAGTTGAGG 3′	746	Okura et al., 2003
	orf8-R:5′AAGTACAGCAGGAGTGAG 3′		

### Adhesion assay

The adhesion assay was performed as described previously (Edwards and Massey, 2011). Briefly, bacteria were cultured for 12 h in MLB at 37°C with shaking at 100 rpm. Washed bacteria (10 μL at approximately 2 × 10^7^cfu mL^−1^; multiplicity of infection [MOI] = 100:1) were added to wells containing a washed coverslip and to wells without a coverslip. Each well contained a confluent layer of Caco-2 cells in 490 μL of DMEM containing 10% FBS without antibiotics. The cells were incubated for 30, 60, 120, and 180 min at 37°C under 5% CO_2_.

### Giemsa staining

After each specific infection, the medium was discarded from the 24-well plates containing glass coverslips, and the wells were washed by the addition of phosphate-buffered saline (PBS; Gibco®, Grand Island, NY, USA), followed by 500 μL of 100% Methanol for 1 min. The supernatant was removed, and Giemsa staining solution was added for 30 min and then discarded. The cells were washed three times with sterile distilled water and left to dry in air, before they were examined under a 100× oil immersion lens.

### Bacterial counts

Twenty-four-well plates without glass coverslips were used to measure the total number of bacteria associated with the cells (adherent and internalized), washed by adding PBS, after that added 500 μL 0.1% Triton X-100 in PBS to each well for 5 min, until cells were fully lysed. The bacteria were counted by plating the liquid suspension, or a diluted suspension if necessary, on LB agar plates containing 3% NaCl using the drop-plate method. Duplicate assays were repeated three times. The results are presented as indices calculated with the formula:

Number of colony-forming units (cfu) of bacterial isolates, adherent and/or internalized in Caco-2 cells/Number of bacteria in the original inoculum (according to the [MOI]) (Benjamin et al., 1995).

### Invasion assay

This assay was the same as the adhesion assay, except that after the bacteria were incubated with cell for different periods, the culture supernatant was removed from each well and replaced with 500 μL of DMEM + 10% FBS supplemented with 100 μg mL^−1^ kanamycin. The plates were incubated at 37°C under 5% CO_2_ for 60 min to kill all extracellular bacteria. The cells were then washed with PBS, stained, lysed, and counted by plating them on LB agar containing 3%NaCl, as described above for the adhesion assay.

### Cytotoxicity assay

The cytotoxicity assay was performed as described previously (Kodama et al., 2007). Briefly, Caco-2 cells were infected with *Vibrio parahaemolyticus* strains at [MOI] = 100:1. The release of lactate dehydrogenase (LDH) into the medium was quantified after 1, 2, 3, and 4 h, with a CytoTox96 Non-Radioactive Cytotoxicity Assay kit (Promega, Madison, WI, USA), according to the manufacturer's instructions. The LDH release (percentage cytotoxicity) was calculated with the following equation: (optical density at 490 nm [OD_490_] after experimental release–[OD_490_] after spontaneous release/[OD_490_] after maximum release—[OD_490_] after spontaneous release) × 100. Spontaneous release is the amount of LDH released from the cytoplasm of uninfected Caco-2 cells, and the maximum release is the amount of LDH released by the total lysis of uninfected Caco-2 cells.

### Apoptosis and dead cell detection

Apoptotic cells were detected with the Annexin V-FITC Apoptosis Detection Kit (BD Biosciences, San Diego, CA, USA), according to the manufacturer's instructions. Briefly, the cells were seeded in 24-well plates, infected with bacteria at [MOI] = 100:1, and incubated for 1, 2, 3, and 4 h after infection. Approximately 1 × 10^5^ cells were harvested with a cell collector, centrifuged (250 × g for 5 min), washed twice with PBS, and stained with Annexin V-FITC and PI, according to the manufacturer's instructions. Fluorescence was detected by a flow cytometer with FACSAria flow cytometer using the FACSDiva software (Becton-Dickinson Immunocytometry System, BDIS, San Jose, CA, USA) (Pan et al., 2013).

### Measurement of reactive oxygen species (ROS) assay

Intracellular ROS levels were determined using the fluorescent marker 2′,7′-dichlorodihydrofluorescein diacetate (DCFH-DA; S0033; Beyotime, China), according to the manufacturer's instructions. Briefly, the cells were seeded in 24-well plates and infected with bacteria at [MOI] = 100:1, incubated for 1, 2, 3, and 4 h after infection, harvested with a cell collector, centrifuged (250 × g for 5 min), washed twice with PBS, and incubated with 10 μM DCFH-DA for 20 min at 37°C. Fluorescence intensity was analyzed with a FACSAria flow cytometer using FACSDiva software (Becton-Dickinson Immunocytometry System) (Itoh et al., 2010).

### Analysis of intracellular Ca^2+^ concentrations, nitric oxide (NO), intracellular pH, and mitochondrial membrane potential (MMP)

Briefly, cells were seeded in 24-well plates, infected with bacteria at [MOI] = 100:1, and incubated for 1, 2, 3, and 4 h after treatment the cells were harvested with a cell collector, centrifuged (250 × g for 5 min), and washed twice with PBS.

To measure the intracellular concentrations of calcium ions (Ca^2+^), cells were incubated with 5 μM Fluo-3 AM (S1056; Beyotime, China) for 15 min at 37°C. To determine the intracellular production of NO, cells were incubated with 5 μM DAF-FM DA (S0019; Beyotime, China) for 20 min at 37°C. The collected cells were incubated with 5 μM BCECF AM (S1006; Beyotime, China) for 30 min at 37°C to determine the intracellular pH (Chow and Hedley, 2001), or with 10 μM JC-1 (C2006; Beyotime, China) for 20 min at 37°C in the dark to determine MMP (Pan et al., 2013). Fluorescence intensity was analyzed with a FACSAria flow cytometer using the FACSDiva software (Becton-Dickinson Immunocytometry System).

### Microstructural changes in infected cells

Caco-2 cells were incubated with bacteria at [MOI] = 100:1 for 1 and 3 h, and then prepared for transmission electron microscopy (TEM) analysis, as described previously (Zhang et al., 2012). The samples were examined with a Philips Tecnai™ transmission electron microscope.

### Statistical analyses

All statistical analyses were performed using (GraphPad Prism, San Diego, CA, USA).

## Results

### Adhesion of *vibrio parahaemolyticus* to cell monolayers

**Figure 1:**
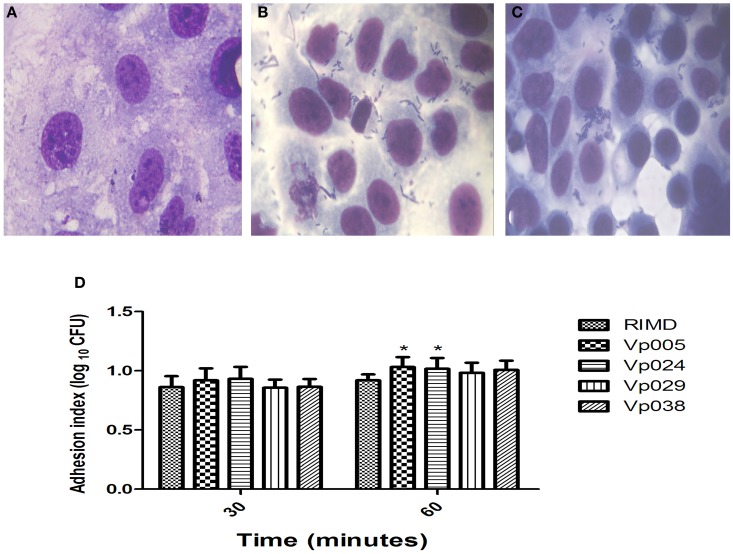
**Adhesion index of *Vibrio parahaemolyticus* in Caco-2 cells**. Caco-2 cells were infected with *Vibrio parahaemolyticus* strains at [MOI] = 100:1. The reported values are the means ± SEM of three independent experiments (*n* = 6). **(A)** Uninfected Caco-2 cells were used as the control. **(B,C)** Caco-2 cells infected with RIMD [MOI] = 100:1 at 30 and 60 min, respectively (100×, oil immersion). **(D)** Means adhesion indices differed statistically significantly from one another on Two-Way analysis of variance (ANOVA) (^*^P < 0.05).

Our results indicate that the bacterial adherence was high a short time (30 min) after infection (Figures 1A–C). There were no significant differences between the strains after 30, 120, and 180 min after infection, but there were significant differences between RIMD and both Vp005 and Vp024 only 60 min after infection (Figure 1D) and (Table 3).

**Table 3:** **Adhesion and Invasion Indices**.

**Strain**	**Virulent genes**				**Adhesion index**				**Invasion index**			
	***tlh***	***tdh***	***trh***	***Orf8***	**30**	**60**	**120**	**180**	**30**	**60**	**120**	**180**
RIMD	+	+	−	+	0.86±0.09	0.91±0.05	1.09±0.04	1.15±0.01	0	0	0	0.61±0.06
Vp005	+	+	−	−	0.91±0.1	1.03±0.08	1.11±0.03	1.12±0.01	0	0.08±0.19	0.16±0.25	0.56±0.05
Vp024	+	+	−	+	0.93±0.1	1.01±0.09	1.11±0.05	1.14±0.02	0.05±0.13	0.21±0.24	0.58±0.06	0.52±0.04
Vp029	+	−	−	−	0.85±0.06	0.98±0.08	1.08±0.05	1.11±0.02	0	0	0.56±0.07	0.64±0.03
Vp038	+	+	−	+	0.86±0.06	1.0±0.07	1.03±0.07	1.13±0.01	0	0.12±0.18	0.54±0.03	0.53±0.04

### Invasion and replication of *vibrio parahaemolyticus* inside host cells

**Figure 2:**
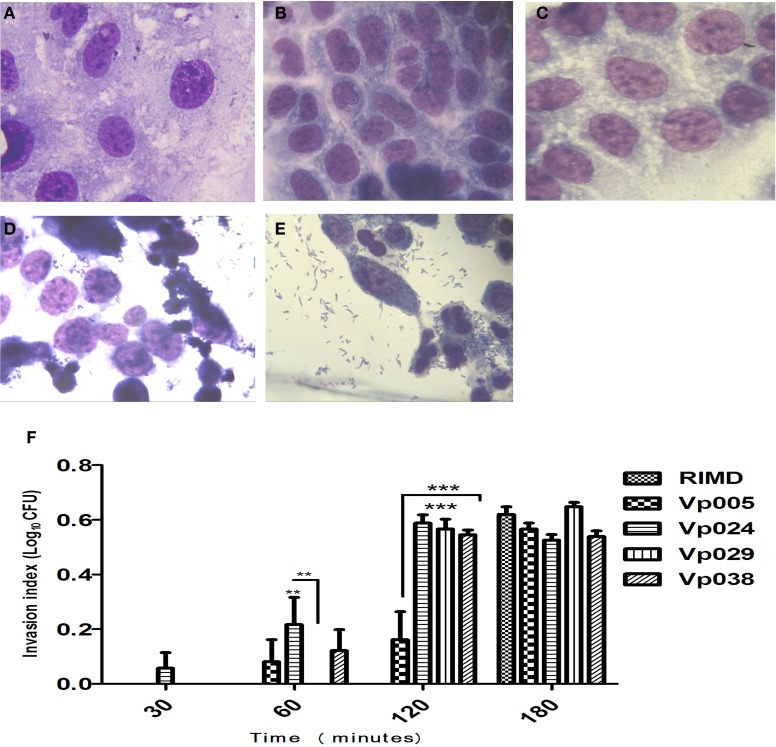
**Invasion index of *Vibrio parahaemolyticus* in Caco-2 cells**. Caco-2 cells were infected with *Vibrio parahaemolyticus* strains. The reported values are the means ± SEM of three independent experiments (*n* = 6). **(A)** Uninfected Caco-2 cells were used as the control. **(B–E)** Caco-2 cells infected with RIMD [MOI] = 100:1 at 30, 60, 120, and 180 min, respectively (100X, oil immersion). **(F)** Means invasion indices differed statistically significantly from one another on Two-Way ANOVA (^**^P < 0.01; ^***^P < 0.001).

No bacteria were recovered from the RIMD-treated cells at 30, 60, or 120 min after infection, but bacteria were recovered at 180 min (Figures 2A–E). The same observation was made of all cells infected with Vp005, Vp029, or Vp038 after 30 min, but no bacteria were recovered from Vp029-treated cells at 60 min. There were no significant differences between any of the strains after 180 min, but the significant differences were observed between RIMD and Vp024 at two time points (60 and 120 min) and between RIMD and Vp029, and between RIMD and Vp038 at 120 min. There were also significant differences between Vp005 and Vp024, Vp005 and Vp029, and Vp005 and Vp038 at the same time (120 min), and a significant difference between Vp024 and Vp029 at 60 min after infection (Figure 2F) and (Table 3).

**Figure 3:**
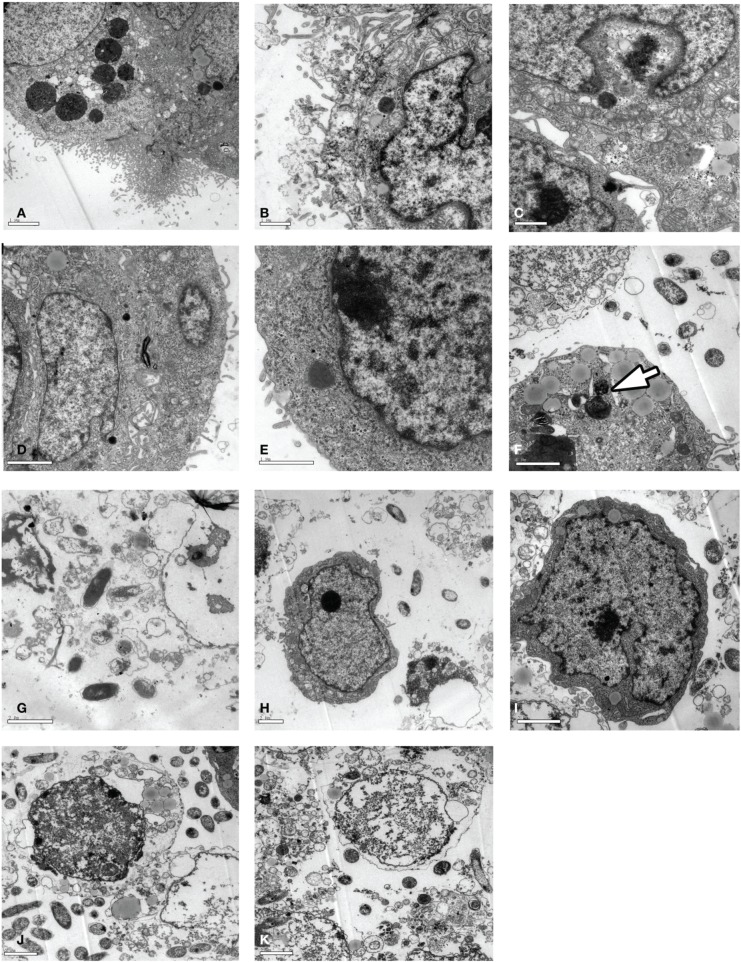
**TEM analysis of Caco-2 cells infected with *Vibrio parahaemolyticus***. Caco-2 cells were infected with strain (RIMD, Vp005, Vp024, Vp029, or Vp038) at [MOI] = 100:1 and incubated for 1 or 3 h. The cells were collected for TEM examination. **(A–E)** Cells infected with *Vibrio parahaemolyticus* RIMD, Vp005, Vp024, Vp029, and Vp038, respectively and incubated for 1 h; appear as bacteria surrounded by cell membrane. **(F)** Arrow indicates replicating bacterium inside a Caco-2 cell. **(G–K)** Cells infected with *Vibrio parahaemolyticus* RIMD, Vp005, Vp024, Vp029, and Vp038, respectively and incubated for 3 h; destruction of cells and changes in their character, and the appearance of bacteria. Scale bar = 2 μm.

Surprisingly, *Vibrio parahaemolyticus* strains were able to replicate inside the non-phagocytic cells (host cells), as observed after 1 h, which was confirmed by TEM examination of the bacterially infected cells (Figures 3A–F).

**Figure 4:**
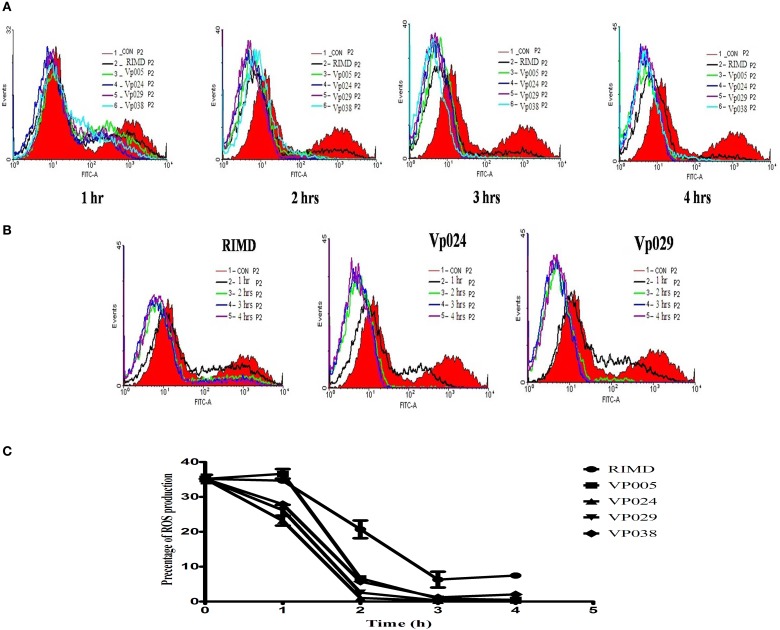
**ROS levels in Caco-2 cells**. ROS levels were determined in Caco-2 cells by staining with DCFH-DA, and flow cytometric analysis. **(A)** ROS levels in uninfected cells (control) and in cells infected with all strains at each specific time points. **(B)** ROS levels in uninfected cells and cells infected with RIMD, a pandemic strain (Vp024), or a non-pandemic strain (Vp029) at different time points. **(C)** Chart showing the percentages of ROS levels. The reported values are the means ± SEM of three independent experiments.

Furthermore, surprisingly, the intracellular ROS levels in Caco-2 cells infected with the *Vibrio parahaemolyticus* strains for different times (1, 2, 3, or 4 h after infection) were lower than those in the uninfected (control) cells (Figure 4). Therefore, several parameters related to ROS, including NO, Ca^2+^, pH, and MMP, were examined.

**Figure 5:**
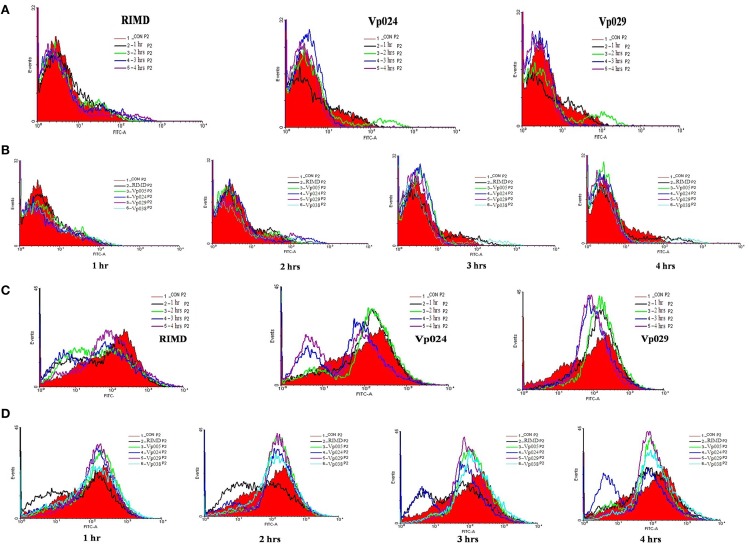
**Intracellular NO and Ca^2+^ levels in the infected Caco-2 cells**. Detection of NO and Ca^2+^ in Caco-2 cells, stained with DAF-FM DA and Fluo-3 AM, respectively and analyzed with flow cytometry **(A)** NO levels in uninfected cells and cells infected with RIMD, a pandemic strain (Vp024) or a non-pandemic strain (Vp029) at different time points. **(B)** NO levels in uninfected cells (control) and in cells infected with each strains at each specific time point. **(C)** Ca^2+^ levels in uninfected cells and cells infected with RIMD, a pandemic strain (Vp024), and a non-pandemic strain (Vp029) at different time points. **(D)** Ca^2+^ levels in uninfected cells (control) and in cells infected with each strain at each specific time point.

All strains clearly increased Ca^2+^ levels in the cells after infection. In contrast, cellular NO production decreased in the cells after infection by all the strains investigated (Figure 5). The intracellular pH of the cells infected with each strain was slightly lower than that of the control group and decreased slightly with the increase in time after infection. MMP of the infected cells increased, but then decreased 3 h after infection with each strain of *Vibrio parahaemolyticus*. This was clearly observed in the cells infected with Vp029 or Vp038, whereas MMP decreased only slightly in cells infected with Vp005 or Vp0024. However, MMP was stable in cells infected with RIMD (Table 4).

**Table 4:** **Results of pH and MMP assays**.

**Time (h)**	**Ctrl**		**RIMD**		**Vp005**		**Vp024**		**Vp029**		**Vp038**	
	**pH**	**MMP**	**pH**	**MMP**	**pH**	**MMP**	**pH**	**MMP**	**pH**	**MMP**	**pH**	**MMP**
1	1.32:1	1.52:1	1.29:1	1.84:1	1.22:1	1.56:1	1.15:1	1.99:1	1.23:1	2.77:1	1.16:1	1.75:1
2	1.32:1	1.52:1	1.27:1	2.38:1	1.19:1	4.44:1	1.20:1	1.99:1	1.20:1	5.07:1	1.13:'1	1.80:1
3	1.32:1	1.52:1	1.22:1	1.70:1	1.13:1	5.83:1	1.14:1	2.30:1	1.11:1	6.45:1	1.12:1	5.29:1
4	1.32:1	1.52:1	1.20:1	1.70:1	1.09:1	5.31:1	1.00:1	2.29:1	0.99:1	5.29:1	1.12:1	3.93:1

### Cell damage caused by *vibrio parahaemolyticus*

Initial infection at [MOI] = 100:1 and the cytotoxicity assay demonstrated that all the *Vibrio parahaemolyticus* strains caused cellular morphological changes, followed by cell lysis approximately 4 h after infection, as shown with Giemsa staining (Figures 2D,E) and comfirmed with TEM (Figures 3G–K) at 3 h after infection, the number of bacteria increased, accompanied by a dramatic reduction in cell numbers, coincident with cell lysis.

### *Vibrio parahaemolyticus* is cytotoxic to host cells

**Figure 6:**
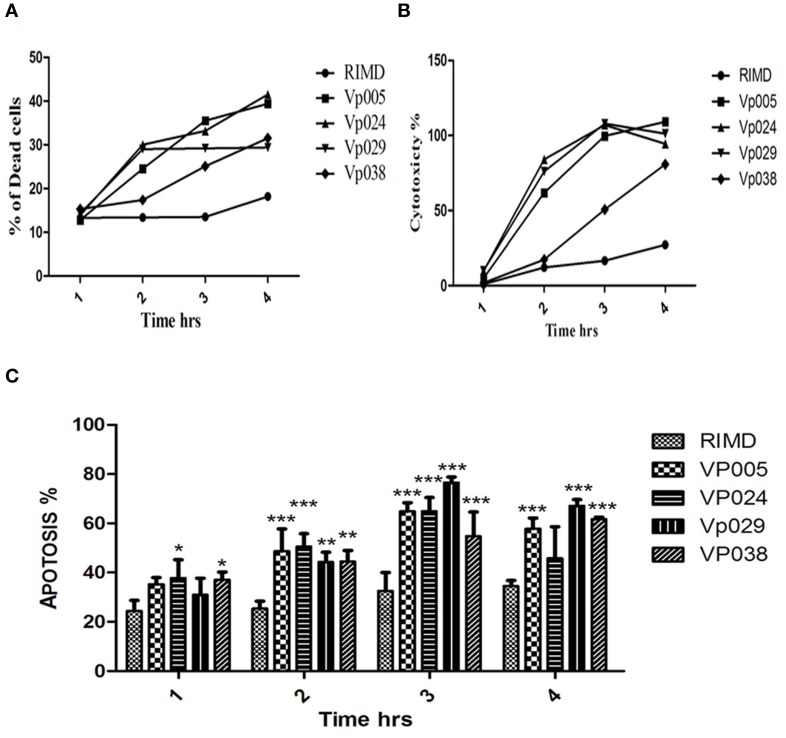
**Cytotoxic effects of *Vibrio parahaemolyticus* on Caco-2 cells**. Caco-2 cells were infected with *Vibrio parahaemolyticus* [MOI] = 100:1, the supernatants were collected at specific time points, and the amounts of LDH released were measured. The percentage of dead cells and the type of death (proportion of apoptotic cells) were determined using annexin V–FITC and flow cytometry **(A,C)**; and **(B)** LDH release was measured with the CytoTox 96 Non-Radioactive Cytotoxicity Assay (Promega). Percentage cytotoxicity was calculated using the formula: (test LDH release—spontaneous release)/maximal release. Test LDH release is the LDH released after infection with different *Vibrio parahaemolyticus* strains; spontaneous release is the baseline cell LDH release, with no bacterial infection; and maximal LDH release is the release of LDH when cells are lysed with lysis solution. The data are the means of three independent experiments ± SEM, and statistically significant differences between the mean values were detected with Two-Way ANOVA (^*^P < 0.05; ^**^P < 0.01; ^***^P < 0.001).

We analyzed the cellular contents released during infection by measuring the levels of cytoplasmic LDH released into the media during the infection of Caco-2 with different strains. This release indicates the degree to which the integrity of the host-cell membrane is compromised at different times. The levels of LDH release caused by strains Vp024 and Vp029, followed by strain Vp005, were higher than that caused by RIMD over the entire time course of the experiment. After 3 h, the levels of LDH release stimulated by Vp024 and Vp029 decreased slightly, in contrast to the continuous increase in the cytotoxicity of the other strains (Figure 6B).

The percentages of apoptotic and dead cells detected 1, 2, 3, and 4 h after infection, showed that almost a third of the cells had died when infected at [MOI] = 100:1. The percentages of dead cells after infection by Vp024 and Vp029, followed by Vp005, were higher than the percentage after RIMD infection, and this result is consistent with the LDH findings. Most of the cells had died by 4 h after infection (Figures 6A,B).

The lowest percentage of apoptotic cells was observed after RIMD infection over the whole time course of the experiment. One hour after infection, the percentage of apoptotic cells among RIMD-infected cells differed significantly from the percentage of apoptotic cells in cells infected with either Vp024 or Vp038. At 2 and 3 h after infection, the percentage of apoptotic RIMD-infected cells differed significantly from those infected with all other strains. The percentage of apoptotic cells induced by RIMD infection differed significantly from the percentages induced by Vp005, Vp029, or Vp038 after 4 h (Figure 6C). The highest percentage of apoptotic cells was observed 3 h after Vp029 infection.

## Discussion

*Vibrio parahaemolyticus* strains encode a number of different virulence factors, including adhesions, TDH, TRH, and two T3SS_s_ (T3SS1 and T3SS2) (Makino et al., 2003). All strains encode T3SS1, which facilitates their survival in the environment, although the natural host(s) of *Vibrio parahaemolyticus* is unknown (Makino et al., 2003; Meador et al., 2007; Paranjpye et al., 2012). These strains also express a number of virulence factors that cause the efficient lysis of the infected host cells and allow the release of valuable nutrients (Burdette et al., 2008). The other (non-T3SS1) virulence factors are found in various combinations in clinical isolates (Meador et al., 2007).

Humans are considered an incidental host of this pathogen, therefore they have played no significant role in the evolution of the virulence factors *Vibrio parahaemolyticus*. However, the animal hosts driving the evolution and persistence of these factors are unknown, although the targets of these toxins and their effectors are evolutionarily conserved (Dean, 2011). Since its discovery in the 1950s, this bacterium has been considered an extracellular pathogen, but recent studies have shown that T3SS2 effectors mediate the bacterial invasion of host cells. Therefore, the roles of T3SS2 in the intracellular lifestyle and pathogenicity of *Vibrio parahaemolyticus* are now being reconsidered (Zhang et al., 2012). Reports of invasive infections in Asian (Hsu et al., 1993) and American (Hally et al., 1995) have also appeared in the literatures. Many studies have focused on how these systems are regulated and how the bacteria maintain strict specificity in the secretion of only designated effectors. Such research will extend our understanding of both this pathogen and other bacterial pathogens.

The present study contributes novel information about the cytotoxic actions of *Vibrio parahaemolyticus* in the intestine, using Caco-2 cell monolayers, which are a functionally faithful and widely accepted *in vitro* model of the human intestinal epithelium. The study suggests a hypothesis about the role of *Vibrio parahaemolyticus* in human disease.

We have shown that *Vibrio parahaemolyticus* is an invasive pathogen and can invade an intestinal cell line, survive, and replicate inside it. There is growing evidence that *Vibrio parahaemolyticus* acts as a persistent microbe and alters ROS production in the host cell to maintain its long-term persistence. It also disrupts the production of NO inside infected cells. Our observations of the direct effects of this pathogen on the cellular physiology, mediated by ROS, and other parameters, together with morphological evidence, indicate that *Vibrio parahaemolyticus* a clear cytotoxic effect on cell monolayer after infection at an [MOI] of 100:1.

Our data suggest that many factors contribute to the invasiveness of *Vibrio parahaemolyticus*. This possibility was first suggested by Akeda and his group (Akeda et al., 2002), who also used Caco-2 cells to show that *Vibrio parahaemolyticus* strains express a cytotoxic factor that acts on cell the cytoskeleton in a calcium-independent fashion.

Our results support the hypothesis that the virulence factors of *Vibrio parahaemolyticus* mediate the mechanisms of its invasion, intracellular replication, and lysis of the infected cells. This is consistent with early observation of the few invasive pathogenic *Vibrio parahaemolyticus* strains (Akeda et al., 1997). Although T3SS2 is a shared by all clinical isolates of *Vibrio parahaemolyticus* and is responsible for their enterotoxicity, it was shown that T3SS2 of *Vibrio* spp. also mediates the invasion of host cell, vacuole formation and the replication of intracellular bacteria (Zhang et al., 2012).

Pathogens manipulate host cell death to facilitate their ability to cause infections (Labbe and Saleh, 2008; Lamkanfi and Dixit, 2010) and this manipulation is achieved with different mechanisms, including apoptosis (a non-inflammatory type of cell death) and necrosis (pro-inflammatory type of cell death) (Festjens et al., 2006). Here, we have demonstrated a mechanism used by *Vibrio parahaemolyticus* to methodically and efficiently induce cell death, and our findings indicate that the cell death caused by this pathogen is independent of the classical apoptotic machinery. This is consistent with the findings that *Vibrio parahaemolyticus* causes non-apoptotic and caspase-independent cell death during infection and that the cell death induced by *Vibrio parahaemolyticus* is associated with autophagy (Burdette et al., 2008).

We observed many different changes in the infected host cells, reflecting the multiple, albeit temporarily orchestrated, mechanisms that culminated in the efficient death of the cells within 4 h of infection. The complete and dramatic changes in the cell structure and its ultimate destruction were undoubtedly caused by changes in the cytoskeleton of the infected host cell. In contrast, other researchers have concluded that TDH exerts its cytotoxic effects both outside and inside the cells and kills the cells via apoptosis (Naim et al., 2001). Finally, we expected that *Vibrio parahaemolyticus* would induce LDH release by causing the death of the non-phagocytic host cells after their invasion, bacterial replication, and cellular nutrients release. The cell death induced by *Vibrio parahaemolyticus* has been investigated in detail, and those earlier studies showed that T3SS1 induces rapid cell death, initiated by acute autophagy in a caspase-independent manner (Burdette et al., 2008).

Generally, to ensure successful micro-organismal infection of host cells (mainly non-phagocytic cells), especially by *Vibrio parahaemolyticus*, and to exert their pathogenic effects, bacteria must protect themselves against host cell oxidative stresses. We found that the intracellular ROS levels of Caco-2 cells were reduced after infection, although elevated ROS is characteristic of the early host innate immune response during the interaction between cells and microbial invaders. Free oxygen radicals are highly toxic to pathogens and are used by the host as a tool to prevent the colonization of tissues by microorganisms (Circu and Aw, 2010). There is direct evidence that a wide variety of micro organisms can limit ROS production in host cells and thus increase the potential for persistent infection by promoting microbial survival within the host cell environment. The strategy used by microorganisms primarily modulates mitochondrially derived ROS (Cirillo et al., 2009). The inhibition of oxygen radicals could facilitate and enhance bacterial colonization by reducing the ROS-mediated host responses to infection. Vp2118 is one of the principal superoxide dismutase- related bacterial proteins (SODs) and is vital in the bacterial anti-oxidative stress responses (Le et al., 2012).

Our results not only extend our knowledge of the cytotoxic effects and enterotoxic properties of *Vibrio parahaemolyticus*. They will also allow us to design further experiments to confirm the proposed mode of the action of its virulence effectors and their role in the pathogenesis of the human gastroenteritis that is associated with the consumption of *Vibrio parahaemolyticus*—contaminated seafood.

## Conclusions

In this study, an *in vitro* cell culture model was established to better understand the pathogenicity of different *Vibrio parahaemolyticus* strains. Our findings demonstrate that all *Vibrio parahaemolyticus* strains have the capacity to adhere to and invade non-phagocytic cells, which are the main targets of this bacterium in the human body. This is especially true of intestinal cells, in which *Vibrio parahaemolyticus* modulates the ROS-based host resistance, promoting its own survival inside the intestinal tract. In this way, it facilitates tissue infection by disrupting the anti-oxidative stress response of the host.

## Author contributions

Shimaa S. El-Malah collection and assembly of the data, manuscript writing, and data analysis; Zhenquan Yang and Qiuchun Li discussion, manuscript revision; Maozhi Hu data analysis and discussion; Zhiming Pan and Xinan Jiao concept and design, data analysis, manuscript revision, and final approval of the manuscript.

### Conflict of interest statement

The authors declare that the research was conducted in the absence of any commercial or financial relationships that could be construed as a potential conflict of interest.
